# Clinical evaluation of fatigue in Japanese patients with Parkinson's disease

**DOI:** 10.1002/brb3.247

**Published:** 2014-07-06

**Authors:** Kenichiro Tanaka, Kenji Wada-Isoe, Mikie Yamamoto, Shugo Tagashira, Yuki Tajiri, Satoko Nakashita, Kenji Nakashima

**Affiliations:** Division of Neurology, Department of Brain and Neurosciences, Faculty of Medicine, Tottori University36-1 Nishi-cho, Yonago, 683-8504, Japan

**Keywords:** Frequency of fatigue, gait disorder, Parkinson Fatigue Scale, relative factors of fatigue, the portable gait rhythmogram

## Abstract

**Background:**

Fatigue is a common nonmotor symptom of Parkinson's disease (PD). Although the causes of fatigue were estimated in the previous reports, fatigue is not fully understood. To determine the frequency of and factors related to fatigue in patients with PD, we carried out clinical assessments in our university hospital.

**Methods:**

We used the Japanese version of the Parkinson Fatigue Scale (J-PFS). The J-PFS was administered to 110 patients with PD, and a cutoff point of 3.3 was used for the diagnosis of fatigue. Subsequently, demographic characteristics, clinical features, and medications utilized were evaluated to elucidate the factors related to fatigue. In particular, we focused on the relationship between fatigue and gait disorder assessed via the portable gait rhythmogram.

**Results:**

The frequency of fatigue in patients with PD was 52.7%. Univariate analysis revealed that factors significantly associated with fatigue were many motor symptoms and nonmotor symptoms. In addition, multivariate analysis revealed that gait disorder and constipation were independent factors related to fatigue. Furthermore, short-step walking and bradykinesia in gait disorder had especially a relationship with fatigue.

**Conclusions:**

More than half of our patients were judged having fatigue. Several factors, including motor and nonmotor symptoms, might be related to fatigue in patients with PD.

## Introduction

### Background

Recently, nonmotor symptoms in patients with Parkinson's disease (PD) have attracted attention. These nonmotor symptoms affect quality of life, institutionalization, and health care costs. Fatigue is one of the nonmotor symptoms of PD, and it might be critical for patient's quality of life. More than half of the patients with PD think that fatigue is one of the three most difficult symptoms (Friedman and Friedman 1993). Nevertheless, fatigue in patients with PD is not commonly identified in the clinical setting. Although the causes of fatigue were estimated in the previous reports, fatigue is not fully understood. In the past, fatigue pathophysiology was discussed, but it was not well characterized. Therefore, the causes of fatigue are divided into several symptoms (Kluger et al. 2013).

### The taxonomies of fatigue

The taxonomies of fatigue vary; in one, fatigue is divided into peripheral fatigue and central fatigue (Chanudhuri and Behan 2000). According to this taxonomy, the fatigue of PD is included in central fatigue. Central fatigue is defined as the failure to initiate and/or sustain attentional tasks and physical activities requiring self motivation. In contrast, peripheral fatigue is fatigue from exercise or physical activity, and is defined as the inability in sustaining a specific force or work rate. In another system, fatigue is divided into mental fatigue and physical fatigue (Okuma 2012). With this classification, it was reported that both mental fatigue and physical fatigue were related to fatigue in patients with PD (Lou et al. 2001).

### Purpose

In this study, we analyzed fatigue in patients with PD, and to determine the frequency of fatigue and factors related to fatigue in PD, we evaluated clinical features, such as demographic characteristics, motor symptoms, nonmotor symptoms, and medications. In particular, we focused on the relationship between fatigue and gait disorder. Gait disorder is one of the most well-known motor symptoms of PD, and it decreases patient's quality of life, but it has not been studied in relation to fatigue fully. In this study, we assessed gait via the portable gait rhythmogram (Mitoma et al. 2010), we evaluated gait from several points of view, and we investigated its relationship with fatigue.

## Methods

### Subjects

The subjects for this study comprised of 110 patients diagnosed with idiopathic PD in the Department of Neurology at Tottori University Hospital between April 2011 and December 2011. The clinical diagnosis of PD was based on the UK PD Society Brain Bank criteria (Gibb and Lees 1988). PD patients diagnosed with dementia according to PD dementia criteria of Movement Disorder Society were excluded beforehand (Poewe et al. 2008).

### Diagnosis of fatigue

We used the Japanese version of the Parkinson Fatigue Scale (J-PFS), which was derived from the Parkinson Fatigue Scale (PFS) in the UK (Okuma et al. 2009). The PFS was the first questionnaire developed for evaluating fatigue in patients with PD, and it consists of 16 items (Brown et al. 2005). Response options were “strongly disagree,” “disagree,” “do not agree or disagree,” “agree,” “strongly agree,” which were scored 1 to 5, respectively. The overall PFS score was calculated as the mean response across all items (range, 1.0–5.0) and the cutoff point of 3.3 was used for the diagnosis of fatigue (an average score of 3.3 or greater indicates the existence of fatigue) (Okuma et al. 2009). The PFS is available in several languages and is used in other countries (Kummer et al. 2011; Hagell et al. 2012). The utility of the J-PFS for fatigue was reported in patients with PD (Okuma 2012).

### Demographic characteristics, clinical features, and medications utilized

We analyzed the demographic and clinical characteristics of each group, including sex, age, age of disease onset defined from medical interviews, PD duration, degree of severity of motor symptoms, nonmotor symptoms (depression, apathy, sleep disturbance, excessive daytime sleepiness, REM sleep behavior disorder [RBD], restless leg syndrome [RLS], orthostatic hypotension, constipation, visual hallucinations, and olfactory dysfunction), medications (total levodopa equivalent dose [LEDs], levodopa, pramipexole, ropinirole, selegiline, and amantadine), and the heart-mediastinum (H/M) ratio of ^123^I-meta-iodobezylguanidine (MIBG) myocardial scintigraphy (Wada-Isoe et al. 2007). We estimated motor symptoms by using the Hoehn–Yahr stage. Nonmotor symptoms were evaluated via questionnaires, including the Geriatric Depression Scale-15 (GDS-15) (Niino et al. 1991), the Apathy Scale (AS) (Okada et al. 1998), the Pittsburgh Sleep Quality Index (PSQI) (Buysse et al. 2003), the Japanese version of the Epworth Sleepiness Scale (JESS) (Takegami et al. 2009), and the REM Sleep Behavior Disorder Screening Questionnaire (RBDSQ) (Stiasny-Kolster et al. 2007). The GDS-15 has been validated for the diagnosis of depression, the AS for apathy, the PSQI for poor sleep, the JESS for excessive daytime sleepiness, and the RBDSQ for RBD. RLS was evaluated using the diagnostic criteria of the International RLS Study Group (Allem et al. 2003). Patients were assessed from medical interviews as positive or negative for orthostatic hypotension, constipation, visual hallucinations, and olfactory dysfunction.

We assessed gait as motor symptoms via the portable gait rhythmogram. The portable gait rhythmogram is a small device that measures the acceleration in trunk movements caused by step-in and kickoff in three dimensions, using accelerometers (Mitoma et al. 2010). It is attached to the patient's waist to evaluate gait for 10 m. The results do not include freezing of gait, since patients actually walk another 2 m before and after the 10 m. We focused on gait acceleration, gait speed, and step length in PD patients. Gait acceleration is the index proportional to floor reaction forces whose unit was measured via G/100 (G: gravity = 9.8 m/s^2^) and the most important end point.

### Statistical analysis

Data analysis was conducted with SPSS for Windows, version 18 (Chicago, IL). The results are presented as medians ± interquartiles range. In univariate analyses, intergroup differences were analyzed with a Mann–Whitney *U-*test, and categorical variances were examined with a χ^2^ test. Correlation analyses were conducted with the Spearman's correlation test. Multivariate analyses were performed via logistic regression analysis. We used the step-wise forward method, and the variables which had statistical significance in univariate analyses were chosen. Regarding gait, we selected gait acceleration because it was the most important end point, and we did not include gait speed and step length because of multicollinearity problems. The goodness-of-fit of the final model was tested by the Hosmer–Lemeshow method. We used a level of 95% (*P* < 0.05) as the criterion for statistical significance.

This study was planned and conducted in accordance with the Declaration of Helsinki. The Ethics Committee of the Tottori University Faculty of Medicine approved the study prior to its implementation.

## Results

### Frequency of fatigue

We were able to administer the J-PFS and to determine the demographic characteristics, clinical features (except for gait), and medications utilized for all 110 PD patients.

The frequency of fatigue was 52.7% (males: 55.1% PD duration 10.0 ± 5.0 years, females: 50.8% PD duration 8.0 ± 3.0 years).

### Demographic features related to fatigue

In univariate analyses, none of the demographic factors, (gender, age, age of disease onset or PD duration) was related to fatigue (Table 1). In analyses calculated via Spearman's correlation test, PD duration was only correlated with the J-PFS score (Table 2).

**Table 1:** Comparison of patients with PD with and without fatigue diagnosed via the J-PFS

Variables	Overall *n* = 110	Without fatigue *n* = 52	With fatigue *n* = 58	*P*-value
J-PFS score	3.2 ± 1.0	2.3 ± 0.5	4.0 ± 0.5	
Demographic factors				
Male:Female	49:61	22:30	27:31	0.703*
Age (years)	70.0 ± 6.5	69.5 ± 6.0	72.0 ± 7.0	0.256
Age of disease onset (years)	61.5 ± 7.0	61.0 ± 6.5	63.0 ± 8.0	0.592
PD duration (year)	8.0 ± 4.5	7.0 ± 4.5	8.5 ± 4.5	0.147
Motor symptoms				
Hoehn–Yahr stage	2.0 ± 0.5	2.0 ± 0.5	3.0 ± 1.0	0.001
Acceleration (gait:G/100)^1^	24.0 ± 7.0	28.0 ± 5.5	20.0 ± 5.0	0.002
Speed (gait:m/min)^1^	56.5 ± 16.0	65.0 ± 16.5	45.0 ± 16.5	<0.001
Step length (gait:cm)^1^	48.5 ± 14.5	55.0 ± 14.0	38.0 ± 14.5	0.001
Nonmotor symptoms				
GDS-15	5.0 ± 2.5	5.0 ± 1.5	7.0 ± 3.0	0.002
AS	17.0 ± 4.0	16.0 ± 3.5	18.0 ± 5.5	0.026
PSQI	6.0 ± 2.0	5.5 ± 2.5	7.0 ± 2.5	0.057
JESS	6.0 ± 4.0	5.0 ± 2.5	8.0 ± 4.0	0.002
RBDSQ	4.0 ± 2.0	3.0 ± 2.0	4.0 ± 2.0	0.040
Restless leg syndrome (*n*)	14 (13%)	4 (8%)	10 (17%)	0.243*
Orthostatic hypotension (*n*)	50 (45%)	19 (37%)	31 (53%)	0.087*
Constipation (*n*)	84 (76%)	34 (65%)	50 (86%)	0.013*
Visual hallucinations (*n*)	33 (30%)	11 (21%)	22 (38%)	0.063*
Olfactory dysfunction (*n*)	40 (36%)	16 (31%)	24 (41%)	0.321*
Early H/M ratio (MIBG)	1.58 ± 0.21	1.68 ± 0.31	1.57 ± 0.15	0.330
Delayed H/M ratio (MIBG)	1.42 ± 0.28	1.60 ± 0.47	1.35 ± 0.16	0.192
Medication				
Total LEDs (mg)	464 ± 173	469 ± 189	462 ± 157	0.297
Dosage of levodopa (mg)	300 ± 100	300 ± 100	350 ± 100	0.003
Dosage of pramipexole (mg)	1.5 ± 1.2	1.0 ± 1.0	1.5 ± 1.2	0.574
Dosage of ropinirole (mg)	4.5 ± 3.5	5.5 ± 4.5	3.0 ± 2.0	0.356
Dosage of selegiline (mg)	2.5 ± 1.5	2.5 ± 1.5	3.8 ± 1.5	0.843
Dosage of amantadine (mg)	150 ± 69	200 ± 125	100 ± 38	0.210

J-PFS, Japanese version of the Parkinson Fatigue Scale; GDS-15, Geriatric Depression Scale-15; AS, Apathy Scale; PSQI, Pittsburgh Sleep Quality Index; JESS, Japanese version of the Epworth Sleepiness Scale; RBDSQ, REM Sleep Behavior Disorder Screening Questionnaire; total LEDs, total levodopa equivalent dose.

Medians ± interquartile range.

Mann–Whitney *U*-test.

* χ^2^ test.

2 75 patients were able to be assessed (without fatigue:with fatigue = 40:35).

**Table 2:** Correlation between the J-PFS and other continuous variables

	Spearman's *ρ*	*P*-value
Demographic factors		
Age (years)	0.182	0.057
Age of disease onset (years)	0.074	0.442
PD duration (year)	0.227	0.017
Motor, nonmotor symptoms		
Hoehn–Yahr stage	0.325	0.001
Acceleration (gait:G/100)^1^	−0.281	0.009
Speed (gait:m/min)^1^	−0.409	<0.001
Step length (gait:cm)^1^	−0.355	0.001
Early H/M ratio (MIBG)	−0.024	0.822
Delayed H/M ratio (MIBG)	−0.101	0.353
GDS-15	0.370	<0.001
AS	0.288	0.002
PSQI	0.251	0.008
JESS	0.269	0.004
RBDSQ	0.233	0.014
Medication		
Total LEDs (mg)	0.155	0.105
Dosage of levodopa (mg)	0.368	<0.001
Dosage of pramipexole (mg)	0.067	0.708
Dosage of ropinirole (mg)	−0.144	0.464
Dosage of selegiline (mg)	0.106	0.511
Dosage of amantadine (mg)	−0.244	0.364

J-PFS, Japanese version of the Parkinson Fatigue Scale; GDS-15, Geriatric Depression Scale-15; AS, Apathy Scale; PSQI, Pittsburgh Sleep Quality Index; JESS, Japanese version of the Epworth Sleepiness Scale; RBDSQ, REM Sleep Behavior Disorder Screening Questionnaire; Total LEDs, total levodopa equivalent dose.

1 75 patients were able to be assessed (without fatigue: with fatigue = 40:35)

### Motor symptoms related to fatigue

In univariate analyses, the severity of Hoehn–Yahr stage, decreased gait acceleration, reduced gait speed, and short-step length were all related to fatigue (Table 1). Of the 110 patients with PD, 75 were able to be assessed for gait via the portable gait rhythmogram. We could not assess 35 patients either because they could not walk by themselves or because we could not contact some of them. The 35 patients who could not be assessed for gait were older and have worse motor function than the 75 patients who could be assessed for gait (Table 3). In analyses calculated via Spearman's correlation test, the severity of Hoehn–Yahr stage, decreased gait acceleration, reduced gait speed, and short-step length were also correlated with the J-PFS score (Table 2).

**Table 3:** Comparison of patients who were assessed for gait and were not

	Assessed for gait, *n* = 75	Not assessed for gait, *n* = 35	*P*-value
Male:Female	33:42	16:19	1.000^1^
Age (years)	69.5 ± 5.5	75.0 ± 7.0	0.007
Age of disease onset (years)	60.0 ± 6.5	66.0 ± 7.0	0.019
PD duration (year)	8.0 ± 4.0	8.5 ± 5.5	0.608
Hoehn–Yahr stage	2.0 ± 0.5	3.0 ± 1.0	0.029

Medians ± interquartile range.

Mann–Whitney *U*-test.

1 χ^2^ test.

### Nonmotor symptoms related to fatigue

In univariate analyses, depression, apathy, excessive daytime sleepiness, REM sleep behavior disorder, and constipation were related to fatigue. The frequencies of sleep disturbance, orthostatic hypotension, and visual hallucinations in the patients with fatigue were higher than those patients without fatigue, but the differences did not reach the statistical significance (Table 1). In analyses calculated via Spearman's correlation test, depression, apathy, sleep disturbance, excessive daytime sleepiness, and REM sleep behavior disorder, were correlated with the J-PFS score (Table 2).

### Relationship factors of dopamine replacement therapy with fatigue

In univariate analyses, only the dosage of levodopa was related to fatigue. Dosages of other medications (LEDs, pramipexole, ropinirole, selegiline, and amantadine) were not related to fatigue (Table 1). In analyses calculated via Spearman's correlation test, the dosage of levodopa was also correlated with the J-PFS score, but other medications were not correlated with fatigue (Table 2).

### Results of multivariate analyses

In multivariate analyses of the patients we could assess for gait, gait acceleration and constipation were found to be independent factors related to fatigue (Table 4).

**Table 4:** Logistic regression model predicting patients with fatigue via the J-PFS (*n* = 75)

	*β*	Odds ratio	95% of CI for odds ratio	*P*-value
Acceleration (gait:G/100)	−0.106	0.899	0.836–0.968	0.005
Constipation	1.408	4.088	1.174–14.235	0.042

J-PFS, Japanese version of the Parkinson Fatigue Scale; CI, confidence interval; GDS-15, Geriatric Depression Scale-15; AS, Apathy Scale; JESS, Japanese version of the Epworth Sleepiness Scale; RBDSQ, REM Sleep Behavior Disorder Screening Questionnaire.

Nagelkerke R2 0.257, Hosmer–Lemeshow test: χ^2^ test = 4.949, *P* = 0.763.

Model contained Hoehn–Yahr stage, gait acceleration, GDS-15, AS, JESS, RBDSQ, constipation, and dosage of levodopa.

## Discussion

### Frequency of fatigue

We calculated the frequency of fatigue in Japanese patients with PD, and more than half had fatigue. Previously, the frequency of fatigue in patients with PD was reported in various areas, and in many, it ranged from 37% to 56% (Fabbrini et al. 2013), so the frequency of fatigue in our patients is similar to that reported in various studies. Although both physical and mental fatigue are involved in PD, the PFS takes mainly physical fatigue into account. It does not account for mental fatigue, so the fatigue of patients with PD in our study placed a disproportionate emphasis on physical fatigue (Falup-Pecurariu 2013). Nevertheless, several nonmotor symptoms were related to fatigue and evaluated via the J-PFS in this study. From the results, it is possible that nonmotor symptoms also contribute to fatigue assessed via the J-PFS.

### Relative factors of fatigue

Past studies have indicated that many factors, including depression, excessive daytime sleepiness, sleep disturbance, motor symptoms, PD duration, and female gender are related to fatigue in PD (Karlsen et al. 1999; Alves et al. 2004; Havlikova et al. 2008; Okuma et al. 2009; Beiske et al. 2010; Valko et al. 2010; Kummer et al. 2011; Metta et al. 2011; Van Dijk et al. 2013). In our study, demographic features, such as gender, age, age of disease onset, and PD duration had little influence on fatigue. These results indicate that fatigue does not have the relation with demographic features. Motor symptoms, such as Hoehn–Yahr stage, gait acceleration, gait speed, and step length were related to fatigue. Gait acceleration was independently related to fatigue in multivariate analysis. In addition, gait speed and step length were also independent relative factors in the multivariate analysis including gait speed or step length instead of gait acceleration (Supporting information). These results indicated that there is a close relationship between gait disorder and fatigue. Although there are some reports describing a relationship between gait disorder and fatigue in PD patients (Garber and Friedman 2003; Rochester et al. 2004, 2006; Rahman et al. 2008), they were not enough because they were not evaluated as to what kind of gait disorder was related to fatigue. Gait disorder in patients with PD has several varieties, such as freezing of gait, short-step walking, festination, bradykinesia, etc. Decreased gait acceleration (floor reaction forces) was related to these gait disorders and was the most important end point. In our study, reduced gait speed and short-step length, as well as decreased gait acceleration, were related to fatigue, so short-step walking and bradykinesia as slow gait cycle walking might be more related to fatigue than festination as fast gait cycle walking. Nonmotor symptoms, such as depression, apathy, excessive daytime sleepiness, RBD, and constipation, were related to fatigue. Among them, constipation was an independent factor related to fatigue. There are some reports that indicate the relationship between other nonmotor symptoms and fatigue, but the relationship between constipation and fatigue has not been reported previously to the best of our knowledge. Therefore, this may be a new insight about fatigue for PD patients. Autonomic nervous systems might be relative factors with fatigue, so we investigated the relationship between fatigue and MIBG myocardial scintigraphy. MIBG myocardial scintigraphy was reported to have a relationship with other nonmotor symptoms, such as olfactory dysfunction and orthostatic hypotension (Oka et al. 2010; Manabe et al. 2011), but it was not related to fatigue in this study. Regarding dopamine replacement therapies, only the dosage of levodopa was related to fatigue, whereas total LEDs was not related. This may be because we could not use other medications, such as dopamine agonists, easily for patient with nonmotor symptoms as the medication that made these symptoms worse (judging from our study, patients with fatigue were likely to have other nonmotor symptoms which got worse by other medications, such as excessive daytime sleepiness, visual hallucination, etc.). In the previous study, the relationship between fatigue and levodopa was reported (Schifitto et al. 2008), but there is not another. These results indicated that medication has little influence on fatigue in PD patients.

### Limitations

There are a few limitations of this study. First, the definition of fatigue in the patients in our study placed a disproportionate emphasis on physical fatigue, because we assessed fatigue via the J-PFS. Next, regarding motor symptoms, the evaluation of patients who could not walk by themselves was not sufficient, since we assessed the motor symptoms, except for Hoehn–Yahr stage, via portable gait rhythmography. In the gait assessment, we could not evaluate freezing of gait. Finally, we did not study a true random sample of patients with PD, since our study population consisted of only those patients who visited our hospital.

## Conclusion

Many PD patients had fatigue as reported in the past, and various factors, such as motor symptoms and nonmotor symptoms, were related to fatigue, but it was not clear which of these symptoms related to fatigue caused fatigue or if these symptoms occurred from fatigue. However, these symptoms had no small effect on fatigue, so we should pay attention to fatigue in clinical settings.

## Supporting Information

**Table S1.** Logistic regression model predicting patients with fatigue via the J-PFS (*n* = 75).

## References

[b1] Allem RP, Picchietti D, Hening WA, Trenkwalder C, Walters AS, Montplaisi J (2003). Restless legs syndrome: diagnostic criteria, special considerations, and epidemiology. A report from the restless legs syndrome diagnosis and epidemiology workshop at the National Institutes of Health. Sleep Med.

[b2] Alves G, Wentzel-Larsen T, Larsen JP (2004). Is fatigue an independent and persistent symptom in patients with Parkinson disease. Neurology.

[b3] Beiske AG, Loge JH, Hjermstad MJ, Svensson E (2010). Fatigue in Parkinson's disease: frequency and associated factors. Mov. Disord.

[b4] Brown RG, Dittner A, Findley L, Wessely SC (2005). The Parkinson fatigue scale. Parkinsonism Relat. Disord.

[b5] Buysse DJ, Reynold IIICF, Monk TH, Berman SR, Kupfer DJ (2003). The Pittsburgh Sleep Quality Index: a new instrument for psychiatric practice and research. Psychiatry.

[b6] Chanudhuri A, Behan PO (2000). Fatigue and basal ganglia. J. Neurol. Sci.

[b7] Fabbrini G, Latorre A, Suppa A, Bloise M, Frontoni M, Berardelli A (2013). Fatigue in Parkinson's disease: motor or non-motor symptoms. Parkinsonism Relat. Disord.

[b8] Falup-Pecurariu C (2013). Fatigue assessment of Parkinson's disease patient in clinic: specific versus holistic. J. Neural. Transm.

[b9] Friedman J, Friedman H (1993). Fatigue in Parkinson's disease. Neurology.

[b10] Garber CE, Friedman JF (2003). Effect of fatigue on physical activity and function in patients with Parkinson's disease. Neurology.

[b11] Gibb WR, Lees AJ (1988). The relevance of the Lewy body to the pathogenesis of idiopathic Parkinson's disease. J. Neurol. Neurosurg. Psychiatry.

[b12] Hagell P, Rosblom T, Palhagen S (2012). A Swedish version of the 16-item Parkinson fatigue scale (PFS-16). Act. Neurol. Scand.

[b13] Havlikova E, Rosenberger J, Nagyova I, Middel B, Dubayova T, Gdovinova Z (2008). Clinical and psychosocial factors associated with fatigue in patients with Parkinson's disease. Parkinsonism Relat. Disord.

[b14] Karlsen K, Larsen JP, Tandberg E, Jørgensen K (1999). Fatigue in patients with Parkinson's disease. Mov. Disord.

[b15] Kluger BM, Krupp LB, Enoka RM (2013). Fatigue and fatigability in neurologic illnesses. Neurology.

[b16] Kummer A, Scalzo P, Cardoso F, Teixeira A (2011). Evaluation of fatigue in Parkinson's disease using the Brazilian version of Parkinson's fatigue scale. Acta Neurol. Scand.

[b17] Lou J-S, Kearns G, Oken B, Sexton G, Nutt J (2001). Exacerbated physical fatigue and mental fatigue in Parkinson's disease. Mov. Disord.

[b18] Manabe Y, Fujii D, Kono S, Sakai Y, Tanaka T, Narai H (2011). Systemic blood pressure profile correlates with cardiac ¹²³I-MIBG uptake in patients with Parkinson's disease. J. Neurol. Sci.

[b19] Metta V, Logishetty K, Martinez-Martin P, Gage HM, Schartau PE, Kaluarachchi TK (2011). The possible clinical predictors of fatigue in Parkinson's disease: a study of 135 patients as part of International Nonmotor Scale Validation Project. Parkinsons Dis.

[b20] Mitoma H, Yoneyama M, Orima S (2010). 24-hour recording of parkinsonian gait using a portable gait rhythmogram. Intern. Med.

[b21] Niino N, Imaizumi T, Kawakami N (1991). A Japanese translation of the Geriatric Depression Scale. Clin. Gerontol.

[b22] Oka H, Toyoda C, Yogo M, Mochio S (2010). Olfactory dysfunction and cardiovascular dysautonomia in Parkinson's disease. J. Neurol.

[b23] Okada K, Kobayashi S, Aoki K, Suyama N, Yamaguchi S (1998). Assessment of motivational loss in poststroke patients using the Japanese version of Starkstein's Apathy scale. Jpn. J. Stroke.

[b24] Okuma Y (2012). Fatigue and weight loss in Parkinson's disease. Brain Nerve.

[b25] Okuma Y, Kamei S, Morita A, Yoshii F, Yamamoto T, Hashimoto S (2009). Fatigue in Japanese Patients with Parkinson's disease: A Study Using Parkinson Fatigue Scale. Mov. Disord.

[b26] Poewe W, Gauthier S, Aarsland D, Leverentz JB, Barone P, Weintraub D (2008). Diagnosis and management of Parkinson's disease dementia. Int. J. Clin. Pract.

[b27] Rahman S, Griffin HJ, Quinn NP, Jahanshahi M (2008). The factors that induce or overcome freezing of gait in Parkinson's disease. Behav. Neurol.

[b28] Rochester L, Hetherington V, Jones D, Nieuwboer A, Kwakkel G, Van Wegen E (2004). Attending to the task: interference effects of functional tasks on walking in Parkinson's disease and the roles of cognition, depression, fatigue, and balance. Arch. Phys. Med. Rehabil.

[b29] Rochester L, Jones D, Hetherington V, Nieuwboer A, Willems A, Kwakkel G (2006). Gait and gait-related activities and fatigue in Parkinson's disease: What is the relationship?. Disabil. Rehabil.

[b30] Schifitto G, Friedman JH, Oakes D, Shilman L, Comella CL, Marek K (2008). Fatigue in levodopa-naïve subjects with Parkinson disease. Neurology.

[b31] Stiasny-Kolster K, Mayer G, Schäfer S, Möller JC, Heinzel-Gutenbrunner M, Oertel WH (2007). The REM sleep behavior disorder screening questionnaire - a new diagnostic instrument. Mov. Disord.

[b32] Takegami M, Sugimoto Y, Wakita T, Noguchi H, Chin K, Kadotani H (2009). Development of a Japanese version of the Epworth Sleepiness Scale (JESS) based on item response theory. Sleep Med.

[b33] Valko PO, Waldvogel D, Weller M, Bassetti CL, Held U, Baumann CR (2010). Fatigue and excessive daytime sleepiness in idiopathic Parkinson's disease differently correlate with motor symptoms, depression, and dopaminergic treatment. Eur. J. Neurol.

[b34] Van Dijk JP, Havlikova E, Rosenberger J, Nagyova I, Skorvanek M, Gdovinova Z (2013). Influence of disease severity on fatigue in patients with Parkinson's disease is mainly mediated by symptoms of depression. Eur. Neurol.

[b35] Wada-Isoe K, Kitayama M, Nakaso K, Nakashima K (2007). Diagnostic markers for diagnosing dementia with Lewy bodies: CSF and MIBG cardiac scintigraphy study. J.Neurol. Sci.

