# Supplementary material for: Clinical evaluation of fatigue in Japanese patients with Parkinson's disease
Source: Brain Behav. 2014 Jul 6;4(5):643–9. doi: 10.1002/brb3.247 (PMC4107378; doi:10.1002/brb3.247)
Supplement: Supplementary file 1 — Table S1. Logistic regression model predicting patients with fatigue via the J-PFS (n = 75). [file brb30004-0643-SD1.docx]

Supporting Information

Table. Logistic regression model predicting patients with fatigue via the J-PFS (n=75)

1. The analysis including speed instead of acceleration

|  | β | Odds ratio | 95% of CI for odds ratio | P-value |
| --- | --- | --- | --- | --- |
| Speed (gait:m/min)  Constipation | -0.044  1.320 | 0.957  3.743 | 0.932-0.983  1.046-13.397 | 0.001  0.043 |

Nagelkerke R2 0.302, Hosmer-Lemeshow test: χ^2^ test = 3.884, p=0.867

Model contained Hoehn-Yahr stage, gait speed, GDS-15, AS, JESS, RBDSQ, constipation, and dosage of levodopa.

2. The analysis including step length instead of acceleration

|  | β | Odds ratio | 95% of CI for odds ratio | P-value |
| --- | --- | --- | --- | --- |
| Step length (gait:cm)  Constipation | -0.047  1.348 | 0.954  3.851 | 0.926-0.984  1.086-13.655 | 0.002  0.037 |

Nagelkerke R2 0.275, Hosmer-Lemeshow test: χ^2^ test = 4.390, p=0.820

Model contained Hoehn-Yahr stage, step length, GDS-15, AS, JESS, RBDSQ, constipation, and dosage of levodopa.

(Abbreviation) J-PFS: Japanese version of the Parkinson Fatigue Scale, CI: confidence interval, GDS-15: Geriatric Depression Scale-15, AS: Apathy Scale, JESS: Japanese version of the Epworth Sleepiness Scale, RBDSQ: REM Sleep Behavior Disorder Screening Questionnaire
